# The Pulmonary Circulation in the Single Ventricle Patient

**DOI:** 10.3390/children4080071

**Published:** 2017-08-07

**Authors:** Amanda Hauck, Nicolas Porta, Steve Lestrud, Stuart Berger

**Affiliations:** Divisions of Neonatology, Pediatric Pulmonary Medicine and Pediatric Cardiology, Northwestern University Medical School, Lurie Children’ Hospital, 225 E, Chicago Avenue, Chicago, IL 60622, USA; ahauck@luriechildrens.org (A.H.); n-porta@northwestern.edu (N.P.); slestrud@luriechildrens.org (S.L.)

**Keywords:** single ventricle, Fontan, pulmonary vascular bed

## Abstract

In recent decades, survival of children with complex congenital heart disease has improved considerably. Specifically, children with a variety of congenital heart defects resulting in ‘single ventricle’ physiology can now undergo palliative surgery that allows survival beyond the neonatal period, and in many cases into adulthood, despite having a single functional ventricular pumping chamber supplying both the pulmonary and systemic circulation. Our growing understanding of the functionally univentricular heart has resulted in freedom from Fontan failure of >50% at 25 years post-Fontan. Yet there is still a fair amount of knowledge to be gained, specifically as it relates to the pulmonary circulation in this group of patients. Knowledge gaps relate not only to the pulmonary circulation after Fontan operation, but also at each stage of the single ventricle surgical palliation, including the native physiology prior to any intervention. The pulmonary circulation is affected by multiple issues related to the single ventricle, including specific details of the anatomy unique to each patient, any intervention(s) undertaken, and potential complications such as aortopulmonary collaterals, protein losing enteropathy, plastic bronchitis, venovenous collaterals, pulmonary arteriovenous fistulae, ventricular dysfunction, pulmonary venous stenosis, and more. This chapter will review the current knowledge with regard to the pulmonary circulation in the single ventricle patient, primarily after the Fontan operation. Additionally, it is our hope to help the practitioner assess the pulmonary circulation in the single ventricle patient; we will also discuss the evidence behind and approach to treatment strategies in order to optimize the pulmonary circulation in this complex group of patients.

## 1. History

Congenital heart lesions resulting in single ventricle physiology account for up to 3% of congenital heart lesions, and historically were associated with high mortality in infancy. A variety of heart lesions including hypoplastic left heart syndrome, tricuspid atresia, double inlet left ventricle, and many others are characterized as having a single functional ventricular chamber that must supply both the systemic and the pulmonary circulation. By definition, prior to any interventions, the two circuits exist in parallel rather than in series. This results in obligate arterial desaturation due to mixing of blood and chronic overload of the single ventricle that receives and ejects both circulations. In order to address these conditions, a series of staged surgical palliations are now generally performed for patients with single ventricle physiology, with an ultimate goal of separating systemic venous blood via passive drainage to the pulmonary circulation from the systemic circulation that is ejected by the single ventricle [1].

In 1971, Fontan and Bordeaux first described an approach for the operative treatment of the patient with a functional univentricular heart, separating the systemic and the pulmonary circulation. The concept of the ‘Fontan circulation’ creates a circulation in which the systemic venous return is connected to the pulmonary circuit in the absence of an interposed ventricle, and all shunts (venous, atrial, ventricular, and arterial) are interrupted. This results in near normalization of arterial saturation and elimination of chronic volume overload. This separation of the circulations comes at risk of chronic venous hypertension, congestion of the systemic veins, and decreased cardiac output both at rest and with exercise. Historically, several changes to the ‘Fontan approach’ have occurred over the years, including the routine strategy of a bidirectional cavopulmonary anastomosis (usually within the first six months of life) prior to the Fontan operation itself (usually several years later). Further evolution from a ‘classic Fontan’ (an atrial to pulmonary artery connection), has been to what is now considered the optimal approach, the intracardiac lateral tunnel approach, or more recently the extracardiac conduit. There are various reasons for this evolution in strategies and approach. These details will not be discussed in detail but are addressed to some extent later within this chapter.

## 2. Selection of Patients

In 1978, Choussat and Fontan described their important patient criteria necessary to result in a successful Fontan operation [2]. These rules have been modified and refined by many since 1978, but they all reflect that after the Fontan intervention the left atrial pressure and the transpulmonary gradient (TPG = mean PA pressure − LA pressure) must be low. Elevated pulmonary artery pressure is a known risk for early mortality following Fontan that has persisted despite other advances in Fontan management [3,4]. Modern day requirements for a successful Fontan intervention include: unobstructed ventricular inflow, reasonable ventricular function, unobstructed ventricular outflow, unobstructed connection from the systemic venous system into the pulmonary arteries, good sized pulmonary arteries without distortion, a well-developed pulmonary vascular bed, normal pulmonary vascular resistance, and unobstructed pulmonary venous return. In general, TPG < 6 mmHg and PVRi ≤ 3 are associated with acceptable outcomes while in patients at altitude a slightly higher TPG < 8 mmHg is accepted [5,6]. All along in the management of the single ventricle patient, both before and after the actual Fontan operation, management should be aimed toward achieving all these goals. Deviation in any of the above is likely to result in a higher risk of operative mortality or late morbidity or mortality to some degree.

Having described the above, unquestionably, the Fontan concept and its evolution has changed the field such that survival for children with a functionally univentricular heart is not only possible but is expected. The concept of passive cavo-pulmonary flow into the lungs in the absence of a pumping chamber is the principle by which adequate systemic and pulmonary circulation is achieved. This principle has resulted in a reliably high survival rate; but we are far from conquering all of the potential medium and long-term complications. Critical to the success of our management strategy is the protection of the pulmonary vasculature. Variation in the initial single ventricle anatomy and physiology makes it difficult to argue for a ‘one size fits all’ strategy. The presence of pulmonary undercirculation vs. pulmonary overcirculation, systemic obstruction, pulmonary obstruction, pulmonary hypoplasia, and/or pulmonary vein abnormalities are each very different entities that must be recognized with timely and appropriate intervention so that the aforementioned protection and optimization of the pulmonary circulation may occur.

## 3. Current Approach and Strategies Used to Achieve and Optimize the Fontan Circuit

It goes without saying that Fontan circuit creation cannot be achieved at birth because of the inherent elevation of the pulmonary vascular resistance. Additionally, the caval veins and the pulmonary vascular bed are relatively small. Once the pulmonary vascular resistance has diminished, it is typical to entertain a staged approach whereby the superior and inferior systemic venous systems are connected to the pulmonary vascular bed on separate occasions.

In the initial steps of a univentricular anatomy, management must aim for and achieve unrestricted flow from the heart to the systemic circulation, a controlled amount of well-balanced and limited blood flow into the lungs, and an unrestricted blood return to the ventricle. In order to achieve these important entities, interventions in the neonatal period may be necessary. These interventions may include coarctation repair, Damus–Kaye–Stansel, Norwood repair, systemic-to-pulmonary artery shunt, ductal stent, balloon atrial septostomy, or pulmonary artery band. Which of the above interventions are necessary will be dictated by the specific anatomy of the univentricular heart.

At 3–12 months of age, and depending upon the anatomy and physiology, most centers will embark upon a bidirectional cavopulmonary shunt. This procedure was first based on experimental work of Haller and colleagues [7] and first performed by Carlon and colleagues in 1963 for a patient with tricuspid stenosis [8]. Typically, the other sources of pulmonary blood flow (systemic-to-pulmonary artery shunts, antegrade pulmonary blood flow form the heart) are eliminated. Patients remain somewhat cyanotic as inferior caval vein flow will still enter the ventricle and then the systemic circulation. The volume load to the systemic ventricle is slightly less than normal after the bidirectional caval shunt, assuming that there are not additional sources of pulmonary blood flow.

At most centers (with some variability in age, and dictated by the individual clinical situation), the Fontan operation will be performed between one and five years of age. The operation is performed by connection the inferior caval venous return to the pulmonary circuit either via a lateral tunnel connection or an extracardiac conduit. A fenestration may be created between the conduit and the pulmonary atrium. This is done routinely at some centers and in ‘high-risk’ patients at other centers and is undertaken to allow for a residual ‘right-to-left’ shunt in order to increase the preload and cardiac output of the systemic ventricle, and perhaps decreasing systemic venous pressure and systemic venous congestion. This maneuver will be at the expense of a degree of cyanosis but has been shown to reduce post-operative morbidity as well as mortality. The fenestration can be managed with closure at a later date, perhaps in the interventional catheterization lab. Alternatively, the fenestration may be maintained indefinitely to afford the advantages described above for an indefinite period of time.

## 4. Growth and Development of the Pulmonary Vasculature in the Patient with a Univentricular Heart

In almost all patients with a functionally univentricular heart, the growth and development of the pulmonary vasculature will be abnormal. Decreased pulmonary blood flow may be present and may originate in fetal life. Pulmonary blood flow is most certainly less than normal following the bidirectional Glenn operation except in the presence of significant aortopulmonary collateral flow. It is possible for growth of the pulmonary arterial bed after the initial palliation before the bidirectional caval shunt, but an initial systemic-to-pulmonary shunt may cause pulmonary artery distortion as well as a maldistribution of flow to the pulmonary arteries, causing a combination of pulmonary hypoplasia to some areas and vascular disease to others. This latter concept may actually also occur in the absence of a systemic-to-pulmonary artery shunt in a select number of patients. As Gewillig has pointed out, the pulmonary architecture may be further compromised by mechanical obstruction following abnormal connections, ductal constriction, surgical scarring, or external compression [9]. Unfortunately, the bidirectional caval shunt and the Fontan itself create and sustain an abnormal environment for the pulmonary vascular bed and the pulmonary vascular resistance. That abnormal environment includes a desaturated state, longstanding decreased pulmonary blood flow, increasing systemic-to-pulmonary collateral blood flow, lack of pulsatile flow and the inherent endothelial dysfunction, and an absence of periods of high flow and high pressure during exercise—which has the ability to recruit vessels. As the pulmonary vascular resistance increases, all of the above become a vicious cycle inhibiting the growth and development of the pulmonary vasculature.

Allowing for optimal growth and development of the pulmonary vasculature is critical and is probably best achieved before the bidirectional cavopulmonary shunt. The initial palliative procedure is likely the most important determinant of future Fontan well-being. As beautifully discussed by Gewillig, our current strategies provide for only a very limited period of time for this controlled pulmonary overflow and catch up growth to occur, and rather replaces this with a strategy of protection of the single ventricle with a small shunt in order to avoid ventricular volume overload. This difficult balance must take into account the fact that the critically important low pulmonary vascular resistance is determined and maintained by a well-developed vascular bed. If flow to the vascular bed is less than optimal, insufficient growth and development of the pulmonary vascular bed will occur with a resultant higher pulmonary vascular resistance [10]. Therefore, it is conceivable that overprotection of the ventricle at the expense of pulmonary vascular growth may lead to progressive and late severe diastolic dysfunction with increased filling pressures in the Fontan circuit because of chronic underfilling of the pulmonary vasculature and resultant remodeling. The chronic low flow will lead to both pulmonary and systemic vasoconstriction which will perpetuate the vicious cycle and the ultimate failure of entire circuit. Breaking this potential vicious cycle, or better yet, preventing it, is critical.

## 5. Physiology

The physiology of any particular univentricular heart goes hand-in-hand with the anatomic nuances. Key determinants as alluded to above include obstruction to outflow, inflow, or flow across the atrial septum, systemic and pulmonary venous return, pulmonary vascular resistance, atrioventricular valve regurgitation, and systemic ventricular function. For example, with severe systemic outflow obstruction as in hypoplastic left heart syndrome, the neonate is dependent upon right-to-left shunting through a patent ductus arteriosus in order to maintain and support systemic cardiac output. In contrast, if there were a critical obstruction to pulmonary blood flow, a patent ductus arteriosus is necessary for an obligate left-to-right shunt in order to support adequate pulmonary blood flow. Other important issues that can be assessed as per above include the systolic ventricular function, long axis and radial diastolic function, as well as airway mechanics such as structural lung disease or scoliosis. Any and all of these issues have the potential of making the Fontan circuit less than ideal.

On the other hand, if obstruction to pulmonary venous return were present, important pulmonary hypertension may occur. Examples of this scenario could include an atretic or stenotic atrioventricular valve or a restrictive atrial septal defect. Therefore, the optimal physiology of any univentricular heart requires good ventricular function, absence of atrioventricular valve regurgitation, an unrestrictive atrial septal communication, and well balanced systemic and pulmonary blood flow. The latter is determined by the balance of downstream systemic and pulmonary vascular resistances and will be discussed in detail elsewhere.

By way of an additional and important brief physiologic review, particularly as it relates to the Fontan circuit, Rychik (The Relentless Effects of the Fontan Paradox) beautifully describes “the imperfect circulatory physiology” associated with the Fontan as well as its inherent potential complications. Specifically as this relates to the pulmonary circulation, it is the absent ventricular thrust and passive cavo-pulmonary flow which leads to an inherently lower cardiac output than normal with a concomitant inability to deliver normal blood flow into the pulmonary bed. As a result there is decreased ventricular filling and low stroke volume and a chronic state of systemic ventricular volume depletion [5]. This chronic state of volume depletion and inability to increase stroke volume is compounded during exercise or at any time cardiac output might need to increase. The complexities do not stop there as chronotropy may be impaired in this group of patients secondary to the effects of high venous pressure, vascular supply to the sinus node and AV node as well as scarring of the sinoatrial node which can occur for multiple reasons. In any case, chronotropic impairment may further limit cardiac output augmentation in response to an increase in demand. For all the above reasons and others that are likely not yet understood, the overall vascular resistance, both pulmonary and systemic, and ventricular afterload is increased after the Fontan operation. Unlike the normal circulation, blood must traverse the systemic arterial system, the systemic venous system, the Fontan pathway, the pulmonary venous system, and then the systemic ventricle. Endothelial dysfunction [9], as well as potential elevated mesenteric vascular resistance in response to low cardiac output as a compensatory mechanism to shift blood away from non-vital organs [10], may be important and likely contribute to the elevation in vascular resistance.

Also, as elucidated in Rychik’s review, the specific univentricular substrate may not work in favor of the perfect passive cavo-pulmonary flow state. Complexities related to the specific anatomic substrate may also lead to a progressive worsening of the pulmonary circulation over time. Thus, the pulmonary vascular bed may not only be abnormal because of altered pulmonary flow patterns in utero, but can be subjected to deterioration over time after birth either related to surgical interventions or otherwise. What is absolutely clear is that the pulmonary vascular bed is critical in the optimal functionality of passive cavo-pulmonary flow and optimal cardiac output in the Fontan patient. It is also important to note that changes over time, as discussed above, can result in further impairment of the pulmonary circulation. These changes can be etiologically complex and may be related to a previous aorto-pulmonary shunt (pulmonary artery distortion with impairment of pulmonary blood flow and hence impairment of pulmonary vascular bed development), aortopulmonary collateral due to chronic hypoxia, and low cardiac output further impeding passive pulmonary blood flow and adding to ventricular volume overload as well as the factors mentioned above. Potential additional contributing—and perhaps poorly understood—factors include a chronic elevation in venous pressure with passive congestion (a pro-inflammatory state) as a result. Also important are an increased risk for thromboembolism as a result of passive congestion with or without liver dysfunction as well as lymphatic abnormalities which may be related to all of the above. Without question, all of these interrelated issues are important in the Fontan patient. Perhaps inherent is the effect of all these issues on the pulmonary circulation and the untoward effects of a less than optimal pulmonary circuit. The vicious cycle that often perpetuates itself must be broken in order to prevent ongoing and worsening cardiac output, end-organ perfusion, and functionality.

Various forms of collateral vessels can develop in the single ventricle patient. Included are venovenous collateral vessels, aortopulmonary collaterals and pulmonary arteriovenous collateral connections. Venovenous collaterals can develop most commonly after the bidirectional cavo-pulmonary shunt. It is typically a result of the higher venous pressure and the opening of existing small connections or the development of new connections. The concerning result of venovenous connections is cyanosis as a result of venous blood being carried away from the cavopulmonary circuit, away from the lungs and to the heart itself. The typical venovenous connection is a left superior vena cava which becomes larger after the bidirectional caval pulmonary connection and then creates a right-to-left shunt. Venovenous connections can also create cyanosis via a direct connection to the pulmonary veins or left atrium. Aortopulmonary collateral blood vessels cause pulmonary overcirculation. They are likely the result of chronic hypoxia that is present in the majority of single ventricle patients. The degree of aortopulmonary collateral blood vessels are variable and of debatable significance. The benefit of the elimination of these blood vessels, usually by catheter intervention, is also debated frequently. Finally, the development of pulmonary arteriovenous fistulae is another a potential source of cyanosis. It typically is diagnosed in the single ventricle patient with a long-standing cavopulmonary connection but without connection of inferior vena caval blood flow into the pulmonary circuit (i.e., the Fontan connection). It is believed that a factor within the hepatic circulation will prevent the development of these fistulous connections once the Fontan connection adds the ‘factor’ to the pulmonary circulation.

So as an ideal, a low pulmonary vascular resistance is critical and mandatory for an optimally functioning Fontan circuit. In general, as noted above, the pulmonary vascular resistance will control the cardiac output [9]. Gewillig has described that the circuit will run in an “autopilot” mode with the ability for relatively little influence by the clinician. It may be implied that cardiac output in this circumstance is not augmented in the usual manner but rather cardiac output is increased by increasing blood flow into the lungs or by actually bypassing the lungs via a fenestration. Examples of the former strategies might include dilating or stenting obstructed pulmonary arteries. There has been relatively recent interest and experience with the use of pulmonary vasodilators in this setting with an effect that is likely to be modest at best. This will be discussed later. 

Therefore, based on much of what is elucidated above, in order to stack the deck in our favor, management of the single ventricle patient prior to the Fontan itself must be aimed the perfect growth of the pulmonary vasculature. It is the opinion of some that initial pulmonary overcirculation may actually augment the growth of the pulmonary vasculature. As Gewillig has suggested, for example, the optimal balance between a short-lived small shunt in order to minimize ventricular volume overload and preserve cardiac function, and a large longstanding shunt to augment pulmonary growth, is yet to be determined [10]. What is clear, however, is that systemic-to-pulmonary artery shunts do have a potential for distortion of the pulmonary arteries. Perhaps unavoidable, care should be taken to assess pulmonary artery growth and development and intervene early should significant pulmonary artery distortion be found.

## 6. The Pulmonary Vascular Resistance in the Single Ventricle Heart: What We Know about It and What We Can Do about It

Optimizing the pulmonary vascular resistance in the univentricular heart through anatomic strategies from the outset are critical as described above. It is recognized that patients with single ventricle physiology who do not fulfill classic criteria for pulmonary hypertension (mean PAP > 25 mmHg and PVRI > 3) can still have important alterations in pulmonary vascular resistance that place them at risk for poor outcome and may be amenable to pulmonary vasodilatory therapy [11]. Pulmonary vascular resistance is the main determinant affecting the driving force of ventricular preload, especially in patients with apparently good ventricular and valve function [9]. There is growing data on basal pulmonary vascular resistance in patients with single ventricles, on the difficulties in measuring it in a non-pulsatile circulation, and in pharmacologic attempts to manipulate and optimize the pulmonary vascular resistance.

Pulsatile flow is important for shear stress-mediated release of endothelium-derived nitric oxide and for the lowering of the pulmonary vascular resistance by the passive recruitment of capillaries [12,13] Obviously, normal pulsatile flow does not exist in the physiology associated with the bidirectional Glenn or Fontan palliated heart, yet there is limited data on the basal pulmonary vascular resistance or possible mechanisms for abnormalities in vasoreactivity in these patients.

Both animal and human studies have evaluated the effects of non-pulsatile flow on pulmonary vascular resistance and vasoreactivity [13,14,15]. An animal (pig) model of unilateral cavopulmonary anastomosis with non-pulsatile versus minimally pulsatile flow demonstrated a role for pulsatile flow in preventing pulmonary hypertension. While both groups exhibited endothelial cell dysfunction as evidenced by poor vasodilation in response to acetylcholine but not to non-endothelial dependent nitroprusside, the partially pulsatile group attenuated the development of pulmonary hypertension [14]. Khambadkone et al. measured pulmonary vascular resistance and response to iNO in 15 patients at a median of nine years status-post Fontan. The patients with non-pulsatile flow had a baseline PVRI of 2.18 that dropped to 1.82 (*p* < 0.05) after nitric oxide inhalation [15]. Interestingly, the patients with some pulsatility within the Fontan circuit (related mainly to atriopulmonary connections) had no difference in the basal PVRI but did not drop the PVRI in response to nitric oxide inhalation. This data is very suggestive that pulmonary endothelial dysfunction, perhaps related to non-pulsatile flow within the pulmonary circulation, may be important. This study also importantly demonstrated no correlation between the age at follow-up or time since surgery and the elevated PVRI. This might also suggest that the elevated PVRI reflects the early anatomic details and early interventions, that is, whether the anatomy and physiology has been optimized in order to minimize the PVRI. In any case, pulmonary endothelial dysfunction after the Fontan operation is likely multifactorial. It is well known that Fontan physiology leads to flow alterations in the pulmonary bed [16]. For example, there is data suggesting that experimental induction of hyperpolarization of endothelial cells causes cGMP elevation via an NO-dependent mechanism. Included in this process is a redistribution of endothelial cytoskeletal protein, actin, and upregulation of NO synthase gene transcription—all of which may be caused by pulsatile sheer stress. Therefore, reduced pulsatile sheer stress in the Glenn or Fontan may downregulate endothelial NO synthase expression and attenuate endothelial-dependent vasodilatation.

A recent review by Snarr et al. reviewed the limitations of the Fontan physiology and reported on a number of studies that have attempted to characterize molecular level changes responsible for abnormal pulmonary vascular resistance in patients with single ventricles [17]. These studies have shown abnormal expression of endothelial-derived factors in the pulmonary vasculature in patients with failing Fontan physiology. Over-expression of nitric oxide synthase, endothelin-1, and endothelin receptors, as well as reduced expression of bone morphogenetic protein receptor type 2, have all been implicated in vascular remodeling and vasoconstriction in failing single ventricles [18,19]. It is interesting that additional post-mortem studies have shown that many failing Fontan patients have an unusual vascular remodeling phenotype including a small smooth muscle medial layer, smooth muscle cell apoptosis, and eccentric intimal fibrosis [20].

Interestingly and importantly, these studies suggest some important concepts:

1. It will be important to understand the molecular mechanisms involved in the Fontan pulmonary hemodynamics, including and especially the pulmonary vascular resistance.
2. Understanding both the short-term and long-term consequences of this physiology is needed to improve outcomes.
3. Most importantly, identifying the molecular targets that will modulate the abnormal physiology may allow for treatment of a frequent cause of failed single ventricle palliation.

## 7. Pulmonary Vasodilator Therapy—Rationale and Evidence

On the basis of the above information, there seems to be a rationale for attempts at pharmacologic manipulation of the pulmonary vascular resistance in single ventricle patients, with either superior cavopulmonary anastomoses or Fontan, in order to optimize both short-term symptoms and long-term outcomes. Unfortunately, widespread use of pulmonary vasodilators in this population has occurred with only anecdotal success and results from small studies guiding their use.

Pulmonary vasodilator therapy has been used immediately following bidirectional superior cavopulmonary anastomosis in patients with low oxygen saturations immediately post-operatively with some reported success. Agarwal et al. reported on the use of iNO in the post-operative period in patients with elevated Glenn pressure >20 mmHg and showed that most patients were responsive to 20–40 ppm iNO with improvement in Glenn pressure, lower inotrope score and improved oxygenation index [21]. In those patients who did not respond, an anatomic cause for elevated Glenn pressures was found. In contrast, in a group of patients with lower Glenn pressures but systemic saturations <75% and no intrapulmonary shunt, iNO did not seem to have an effect [22]. Further use of pulmonary vasodilators in patients with bidirectional Glenn physiology is reported only in case reports.

Exogenous nitric oxide has been used acutely in post-operative Fontan patients; other pulmonary vasodilator agents have been also used in a more chronic fashion. Use of iNO in the immediate post-operative period following Fontan completion in patients with favorable pre-operative hemodynamics but elevated TPG and CVP following surgery has been shown to significantly reduce TPG and CVP while improving systemic blood pressure [23]. Case reports have also demonstrated success in early use of iNO for patients with significant AVM burden [24,25]. Latus et al. undertook a study to assess the effect of iNO on blood flow dynamics in patients later after Fontan by cardiac MRI measurements [26]. Thirty-three Fontan patients were studied and showed significant changes in pulmonary and systemic blood flow. Specifically, there was a reduction of systemic-to-pulmonary collateral flow and a net increase in systemic blood flow, suggesting the beneficial effects of pulmonary vasodilators on cardiac output, tissue perfusion, and exercise capacity.

This responsiveness to iNO would certainly support the possibility that sildenafil, which limits the breakdown of cGMP produced in response to nitric oxide and has vasodilatory and anti-proliferative effects upon vascular smooth muscle cells, could be a strategy in this patient population.

Sildenafil is the most widely studied pulmonary vasodilator in patients following Fontan palliation. The initial study into its utility to decrease pulmonary vascular resistance and improve exercise capacity in children with Fontan physiology was performed by Giardini et al in 2008 [27]. This study demonstrated an improvement in rest and exercise cardiac index and peak VO2 in response to a single dose of sildenafil. A subsequent study by Van De Bruaene showed an improved cardiac index and stroke volume and a decrease in pulmonary vascular resistance with exercise after a single dose of sildenafil using cardiac MRI [28].

The longer-term response to sildenafil therapy in this population was evaluated in a randomized, double-blind, cross-over trial to evaluate the effect of six weeks of sildenafil on exercise performance and echocardiographic indices of myocardial performance [29,30]. While oxygen consumption was not significantly different between groups after six weeks of therapy, there was a significant improvement in ventilatory efficiency. Significant improvement in echocardiographic markers of ventricular function and cardiac output were also noted [30]. Improvements in response to sildenafil are likely not related to improved ventricular function [31]. Though extremely encouraging, the above studies involve a small number of patients and were designed to assess the short and medium-term effects of sildenafil in the Fontan patients. Data on the long-term effects of sildenafil will be very important in this patient population.

Another pathway for therapeutic intervention could be the endothelin-1 pathway. Endothelin-1 is a peptide that is produced by the endothelial cells and plays an important role in vasoconstriction and cell proliferation and acts through a balance of two endothelin receptors, type A and type B. As discussed above, there has been suggestion of increased levels of endothelin-1 and its receptors in the pulmonary arteries of failed Fontan patients, as well as published reports of elevated plasma levels of endothelin-1 levels in Fontan patients with increased central venous pressure [19,32]. Therefore, since drugs such as bosentan and ambrisentan (endothelin-1 receptor antagonists) have been used in patients with pulmonary hypertension, it may follow that they are of benefit in Fontan patients. Additional enothelin receptor anatgonists include sitaxentan, atrasentan, BQ-123, and zibotentan. The safety of endothelin receptor blocker use in patients with Fontan physiology has been investigated, and may be of importance due to underlying liver dysfunction that is common in these patients. Small studies in the use of bosentan have not found significant short term adverse effects [33,34]. The TEMPO trial was carried out in 2014 in order to assess the effect of bosentan on the exercise capacity of Fontan patients. This randomized, placebo-controlled, double-blind trial showed that after 14 weeks of treatment, the patients treated with bosentan showed significant improvement in peak oxygen consumption, exercise test time, and decreased pro-BNP levels [35]. Shang and colleagues undertook a study to assess the efficacy of bosentan immediately after the Fontan operation in otherwise well patients. In a double-blind, randomized, controlled trial with nine participants, unblinding of the study was undertaken after two years of therapy or placebo. No mortality was noted in either group, however the secondary end-points including 6-min walk distance and NYHA functional class were significantly improved in the bosentan group [34]. Unfortunately, other open-label trials of bosentan in adult Fontan patients have shown no difference in exercise capacity after six months of therapy [36]. Because of the potential confounding variables in such studies, it is important to recognize the need for additional larger scale studies in order to understand the nuances of the Fontan physiology, and the details of any potential benefits to endothelin-1 receptor blocker therapy. The nuances that may be important yet still unknown include the proper selection of patients who will benefit from therapy, long-term effects of these drugs, as well as the optimal age and time for therapy during the course of the patient’s life.

The prostacyclin pathway may also offer a potential therapeutic benefit to the Fontan patient. Prostacyclin is endothelial cell generated and increases production of cyclic AMP which results in vasodilation and also inhibits smooth muscle cell proliferation. Prostacyclin analogues have been used to modulate pulmonary vascular reactivity in pulmonary hypertension. Very few studies have been performed in the Fontan population although a randomized, double-blinded, placebo-controlled trial of inhaled iloprost was undertaken to assess the effect of exercise in Fontan patients [37]. This study showed a significant increase in peak oxygen consumption and peak oxygen pulse after one dose of iloprost. There are no other data on the effect of chronic prostacyclin therapy in the Fontan patient.

An additional interesting and important concept is the role of systemic-to-pulmonary artery collateral flow in the Fontan patient in general, and most certainly in the failing Fontan patient. There is considerable debate on the role of this vasculature in the failing Fontan patient, whether these vessels should be closed, and in what circumstances should that occur [38,39]. We will not discuss that controversy in great detail in this chapter. Suffice it to say that the exact impact of systemic-to-pulmonary collaterals on pulmonary hemodynamics and oxygenation and intrapulmonary shunting is not entirely known. On the one hand these ‘connections’ may be beneficial by allowing a pulsatility to the pulmonary capillary bed, but they may also create high-flow areas with higher vascular resistance in specific areas of the lung. For example, in the paper by Latus [38], the drop in flow within the systemic-to-pulmonary collaterals in response to inhaled nitric oxide could be the result of increase in systemic blood flow due to a reduction in systemic vascular resistance or a redistribution of flow to well-ventilated areas of the lung with a concomitant decrease in intrapulmonary shunting. In any case, these data imply an importance of systemic-to-pulmonary collaterals, and potential benefit from eliminating flow within them. However, a strategy for achieving this is not yet entirely clear. Should they be removed in the catheterization lab routinely in all Fontan patients? Or perhaps should they be removed only in the ‘failing’ Fontan patient? Is this strategy a good long-term approach given that additional new collateral vessels are likely to reform? Is it perhaps more logical to consider a medical approach based on the Latus paper in order to minimize flow through these collateral vessels? We expect that future work will better elucidate the most beneficial approach to systemic-to-pulmonary collaterals including patient selection, timing, and approach.

## 8. Summary

There is little question that the Fontan concept (and its evolution over the years) has been an incredible success, and has enabled the survival of a large number of patients that previously had much less hope. We have learned tremendous amounts from both anatomical and physiological standpoints. In that the group of patients with a functionally univentricular heart represents a very diverse population, with many anatomic variations, there are some important anatomic principles—with regard to inflow to the heart, outflow from the heart, as well pulmonary artery size and distortion—that affect outcomes. In addition to optimizing all of the above to the best of our abilities, protection of the pulmonary vasculature and ensuring a very low pulmonary vascular resistance throughout the lifetime of the Fontan physiology is absolutely critical to our success for every individual patient. Achieving this is challenging in the face of the inherent anatomic challenges of any given patient, the by-definition non-pulsatile flow within the pulmonary vasculature resulting in lack of sheer stress-mediated release of endothelium-derived factors to maintain a low pulmonary vascular resistance, and the likely extensive network of systemic-to-pulmonary artery collaterals. The role of these very complicated and intertwined factors, including the changes at the molecular level, are not entirely clear. In any case, however, a low pulmonary vascular resistance in the Fontan patient is critical. The role of pulmonary vasodilator agents may turn out to be very important in the further palliation of this group of patients. Which agents, when to begin therapy, and how long to treat are yet to be determined.
